# Chemogenetic profiling identifies *RAD17* as synthetically lethal with checkpoint kinase inhibition

**DOI:** 10.18632/oncotarget.5928

**Published:** 2015-09-30

**Authors:** John Paul Shen, Rohith Srivas, Andrew Gross, Jianfeng Li, Eric J. Jaehnig, Su Ming Sun, Ana Bojorquez-Gomez, Katherine Licon, Vignesh Sivaganesh, Jia L. Xu, Kristin Klepper, Huwate Yeerna, Daniel Pekin, Chu Ping Qiu, Haico van Attikum, Robert W. Sobol, Trey Ideker

**Affiliations:** ^1^ Department of Medicine, University of California San Diego, La Jolla, CA, USA; ^2^ Moores Cancer Center, University of California San Diego, La Jolla, CA, USA; ^3^ Bioinformatics and Systems Biology Program, University of California San Diego, La Jolla, CA, USA; ^4^ Department of Pharmacology and Chemical Biology, University of Pittsburgh School of Medicine, Pittsburgh, PA, USA; ^5^ University of Pittsburgh Cancer Institute, Hillman Cancer Center, Pittsburgh, PA, USA; ^6^ Ludwig Institute for Cancer Research, University of California San Diego, La Jolla, CA, USA; ^7^ Department of Toxicogenetics, Leiden University Medical Center, Einthovenweg, Leiden, The Netherlands; ^8^ Department of Genetics, Stanford University School of Medicine, Stanford, CA, USA; ^9^ University of South Alabama Mitchell Cancer Institute, Mobile, Alabama, USA

**Keywords:** RAD17, synthetic lethal, checkpoint kinase inhibitor, biomarker, DNA damage

## Abstract

Chemical inhibitors of the checkpoint kinases have shown promise in the treatment of cancer, yet their clinical utility may be limited by a lack of molecular biomarkers to identify specific patients most likely to respond to therapy. To this end, we screened 112 known tumor suppressor genes for synthetic lethal interactions with inhibitors of the CHEK1 and CHEK2 checkpoint kinases. We identified eight interactions, including the Replication Factor C (RFC)-related protein RAD17. Clonogenic assays in *RAD17* knockdown cell lines identified a substantial shift in sensitivity to checkpoint kinase inhibition (3.5-fold) as compared to *RAD17* wild-type. Additional evidence for this interaction was found in a large-scale functional shRNA screen of over 100 genotyped cancer cell lines, in which *CHEK1/2* mutant cell lines were unexpectedly sensitive to *RAD17* knockdown. This interaction was widely conserved, as we found that *RAD17* interacts strongly with checkpoint kinases in the budding yeast *Saccharomyces cerevisiae*. In the setting of *RAD17* knockdown, CHEK1/2 inhibition was found to be synergistic with inhibition of WEE1, another pharmacologically relevant checkpoint kinase. Accumulation of the DNA damage marker γH2AX following chemical inhibition or transient knockdown of *CHEK1*, *CHEK2* or *WEE1* was magnified by knockdown of *RAD17*. Taken together, our data suggest that CHEK1 or WEE1 inhibitors are likely to have greater clinical efficacy in tumors with *RAD17* loss-of-function.

## INTRODUCTION

Loss-of-function of cell cycle checkpoints is frequent in tumors [1, 2]. Because such tumors have increased reliance on the remaining elements of cell cycle control, targeting the kinases that regulate cell cycle checkpoints has been proposed as an anti-cancer therapeutic strategy [2–5]. Currently, ten selective small molecule inhibitors of the cell-cycle checkpoint kinases CHEK1, CHEK2, or WEE1 have been tested in clinical trials, and many more are in preclinical development [2, 6, 7]. These compounds have shown clinical activity either in combination with DNA damaging chemotherapy or as single agents in several tumor types [2, 4, 5, 8–10]. Recently, WEE1 inhibitors have been explored in combination with CHEK1 and CHEK2 inhibitors [11–13] and also histone deacetylase (HDAC) inhibitors [14].

It has been proposed that checkpoint kinase inhibitors may be most active in tumors with defects in specific aspects of DNA damage repair, including homologous recombination (HR), the Fanconi Anemia pathway, or *TP53* loss-of-function [15–17]. Nonetheless, much remains unknown about the genetic predictors of activity for these compounds. At present, a number of clinical trials involving checkpoint kinase inhibitors are underway [2, 18], but these are being performed without use of biomarker stratification to pre-select patients most likely to respond to therapy. On the other hand, the recent report of a remarkable and possibly curative response to the CHEK1 and CHEK2 (CHEK1/2) inhibitor AZD7762 in a small-cell tumor with *RAD50* mutation illustrates what is possible when a targeted therapy is given to a susceptible tumor [19]. This case highlights the importance of using molecular markers to prospectively identify patients with susceptible tumors so that they can be put on effective therapy.

One general strategy for identifying markers of response to a particular drug is to screen for synthetic-lethal genetic interactions with the drug target [20, 21]. Two genes are said to be ‘synthetic lethal’ if simultaneous disruption of both genes results in cellular death, whereas independent disruption of either gene is tolerated [22]. Cancers with mutations in tumor suppressor genes (TSG) that are synthetically lethal with therapeutic targets such as CHEK1 should be particularly sensitive to inhibition of that target. Consequently, such mutations become markers for selection of patients most likely to respond to targeted therapy. The recent FDA approval of the PARP1 inhibitor olaparib, specifically for ovarian cancer patients harboring *BRCA1* or *BRCA2* mutation, demonstrates the clinical viability of this strategy [23].

Here, we identify synthetic-lethal genetic interactions with CHEK1 in order to stratify tumors with an increased sensitivity to checkpoint kinase inhibition. We identify the human gene RAD17 Homolog (*RAD17*) to be synthetically lethal with both AZD7762 as well as MK-1775, an inhibitor of WEE1. When *RAD17* expression is suppressed, the combination of AZD7762 and MK-1775 shows a potent synergistic toxicity associated with a marked accumulation of γH2AX.

## RESULTS

### Chemogenetic profiling of AZD7762 identifies synthetic lethal interactions with *RAD17* and other DNA repair genes

**Figure 1:**
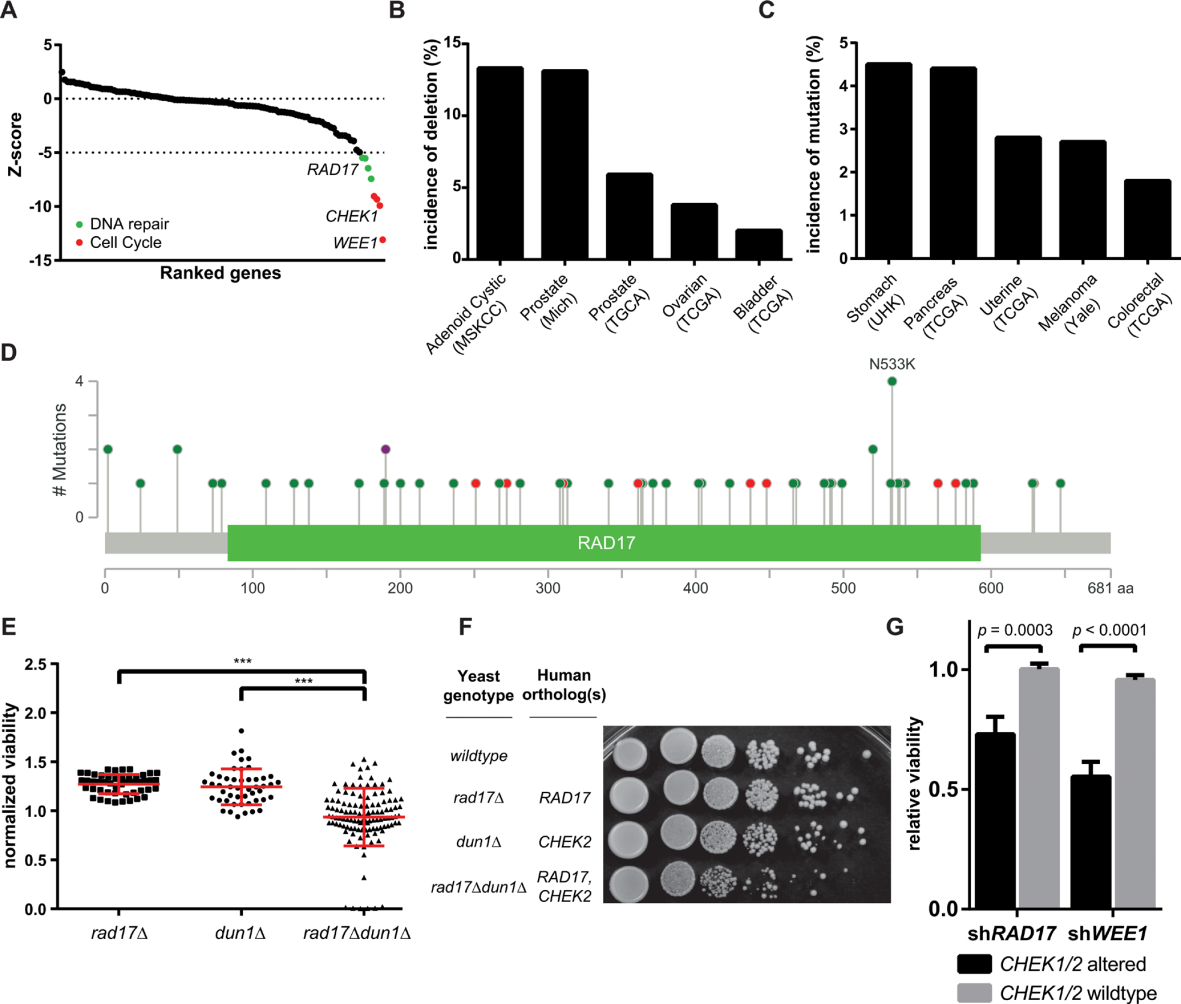
Chemogenetic profiling identifies synthetic lethal interactors with AZD7762 **A.** Rank-ordered results of 112 TSG screened for synthetic lethal interaction with AZD7762, hit genes annotated to DNA repair highlighted in red, hit genes annotated to cell cycle highlighted in green. **B.**, **C.** Incidence of *RAD17* mutation and homozygous deletion in various cohorts (MSKCC - Memorial Sloan Kettering Cancer Center, Mich - University of Michigan, TCGA - The Cancer Genome Atlas, UHK - University of Hong Kong). **D.** Distribution of all missense (green) and truncating (red) *RAD17* mutations reported in TCGA, purple indicates both missense and truncating mutations have been found at a particular nucleotide. Height of bar represents number of mutations observed at a given position. **E.** Synthetic Genetic Array performed in *S. cerevisiae* with *Δrad17*, *Δdun1* and *Δrad17Δdun1* double knockout, each point represents one experimental replicate, *** indicates ANOVA *p* < 0.0001. **F.** Spot dilution assay performed in *S. cerevisiae* with *Δrad17*, *Δdun1* and *Δrad17Δdun1* double knockout. **G.** Cell lines from Project Achilles with *CHEK1/2* mutation or homozygous deletion are more sensitive to knockdown of either *RAD17* or *WEE1* relative to cell lines with wild type *CHEK1/2*, error bars represent +/− SEM, *p* values for *t*-test as indicated.

To identify genes with a synthetic lethal relationship to AZD7762, a chemogenetic screen was performed in HeLa cells against a panel of 112 known or suspected TSG (Supplementary Table 1). Each of the 112 TSG was knocked down with siRNA either in the presence of AZD7762 (high or low dose) or dimethylsulfoxide (DMSO) solvent control. At a stringent cutoff (5 sigma below mean of non-silencing controls), 8 genes were identified for synthetic lethal interaction with AZD7762 (Figure 1A). The top four hits (*WEE1*, *CHEK1*, *CDC6* and *CDC73*) were TSGs with well-known functions related to cell cycle regulation. These included CHEK1 itself, a direct target of AZD7762, as is typical in chemogenetic screens [24]. WEE1, another checkpoint kinase known to complement CHEK1-mediated regulation of cell cycle, also displayed a strong synthetic lethal interaction with AZD7762 [11, 12, 25]. Comparing the AZD7762 screen to a similar chemo-genetic screen with the CHEK1 inhibitor Gö6976 [16], two of the hits with Gö6976, *BRCA2* and *RAD23B* also trended towards being synthetically lethal with AZD7762. Two other hits with Gö6976, *HDAC1* and *HDAC6* were tested but not found to be synthetically lethal with AZD7762. Likely the differences in the AZD7762 and Gö6976 screens is related to inhibition of kinases other than CHEK1. Gö6976 is known to inhibit JAK2 [26], which was been reported to be synergistic with HDAC inhibition [27].

The next four hits with AZD7762 (*BLM*, *RFC1*, *RAD17* and *FZR1*) were all novel interactions involving TSGs functioning in the DNA damage response. Bloom syndrome protein (BLM), a RecQ family DNA helicase, is phosphorylated by both CHEK1 and CHEK2 and is known to participate in HR, telomere maintenance, and DNA replication [28, 29]. Germline mutation in *BLM* is the cause of Bloom syndrome, a rare disease associated with cancer predisposition [28]. Fizzy-related protein homolog (FZR1) activates and regulates substrate specificity of the anaphase-promoting complex/cyclosome (APC/C), and by doing so is thought to regulate multiple cell cycle events including G1/G0 maintenance, initiation of DNA replication and DNA damage response [30]. Replication factor C subunit 1 (RFC1) is a DNA-dependent ATPase known to be involved in clamp loading during DNA replication and repair [31]. First characterized in the fission yeast *Schizosaccheromyces pombe* [32], *RAD17* contains DNA binding motifs similar to *RFC1* and is known to be an important sensor of DNA damage and essential for ATR-mediated cell-cycle arrest in response to DNA damage [33]. RAD17 localizes to areas of DNA damage and recruits the MRN complex to Double Strand Breaks (DSB), promoting HR [34]. In the context of human cancer, it has been shown that depletion of RAD17 sensitizes cancer cell lines to DNA damaging chemotherapy [35], and that down-regulation of RAD17 by certain gain-of-function *TP53* mutations leads to the accumulation of DNA damage [36].

### Loss-of-function of the tumor suppressor *RAD17* is frequent in human cancers

Next, we investigated the incidence of mutation or homozygous deletion in human tumors for each of our hits. Query of the cBioPortal for Cancer Genomics [37, 38] indicated that *RAD17* is the most frequently altered of the four TSGs with a pan-cancer incidence of 1.9% (Supplementary Figure 1). *RAD17* is frequently deleted in adenoid cystic carcinoma (13.3%) and prostate adenocarcinoma (13.1%, 5.9%) and frequently mutated in pancreatic adenocarcinoma (7.0%) and stomach adenocarcinoma (4.5%) (Figure 1B and 1C). Mutations in *RAD17* are spread relatively evenly throughout the 681 amino-acid length of the gene, and 10 of the 58 observed mutations (17.2%) are frameshift, nonsense, or splicing mutations (Figure 1D). This diffuse pattern and frequency of truncating mutations are consistent with *RAD17* functioning as a TSG [39]. A recent pan-cancer analysis of all tumor exomes currently included in The Cancer Genome Atlas (TCGA)[40] found somatic mutations occur in *RAD17* at an overall rate of 1.0% and homozygous deletions at a rate of 0.9%, which, given an annual incidence of approximately 1.7 million new cancer cases in the United States [41], equates to over 31,500 new patients per year with tumors involving *RAD17* loss-of-function.

### *CHEK1/2* - *RAD17* interaction is conserved across species and cancer cell lines

Given the demonstrated utility of cross-species modeling for prediction of chemogenetic interactions [42, 43], we sought to determine if the orthologous genes to *RAD17* and the checkpoint kinases had a synthetic-lethal relationship in the budding yeast *Saccharomyces cerevisiae.* Sequence alignment was performed to identify the following best matches: human *RAD17* with sc*RAD17*; human *CHEK1* with sc*CHK1*, and human *CHEK2* with sc*DUN1* (Supplementary Table 2) [44]. These yeast orthologs were tested in a Synthetic Genetic Array (SGA), in which the *rad17Δdun1Δ* double knockout had significantly smaller colonies relative to either single deletion (Figure 1E). Interestingly, the *rad17Δchek1Δ* interaction did not score as a hit in this in this assay. The *rad17Δdun1Δ* interaction was further tested in a yeast spot dilution assay. The double knockout of *rad17Δdun1Δ* showed less colony formation relative to either *rad17Δ* or *dun1Δ* single deletion or the wild type stain (Figure 1F), confirming that *rad17Δ* and *dun1Δ* have a synthetic lethal relationship.

To determine if the interaction between *CHEK1/2* and *RAD17* was also present across a diverse set of cancer cell lines we analyzed data generated from Project Achilles, a cancer cell-line based functional genomic screen in which over 11,000 genes were knocked down with shRNA in 102 cell lines [45]. Since the majority of these 102 cells lines were profiled for mutations at panel of 1651 genes as part of the Cancer Cell Line Encyclopedia (CCLE) [46] we were able to identify 10 of the 102 as having either mutation or homozygous deletion of *CHEK1* or *CHEK2*. Of note, none of the cell lines had *RAD17* homozygous deletion, and *RAD17* mutation status was not assessed in this dataset. We found that cell lines with disruption of *CHEK1/2* had significantly greater sensitivity to shRNA mediated knockdown of *RAD17* relative to cell lines without *CHEK1/2* alteration (normalized viability 0.73 vs. 1.0, *p* < 0.0003, Figure 1G). Two of the three cell lines most sensitive to *RAD17* knockdown were GP2D and LS411N, both colon cancer cell lines harboring *CHEK1* mutation. The other top hit was Colo704, an ovarian cancer cell line with homozygous deletion of *CHEK2* (Supplementary Table 3). *CHEK1/2* altered cell lines were also more sensitive to knockdown of *WEE1* (normalized viability 0.55 vs. 0.96, *p* < 3.0 x10^−8^, Figure 1G). These data in conjunction with the yeast findings suggest that the interaction between *CHEK1/2* and *RAD17* is general to eukaryotic cells and not specific to a particular genetic background.

### *RAD17* functionally interacts with multiple checkpoint kinases

**Figure 2:**
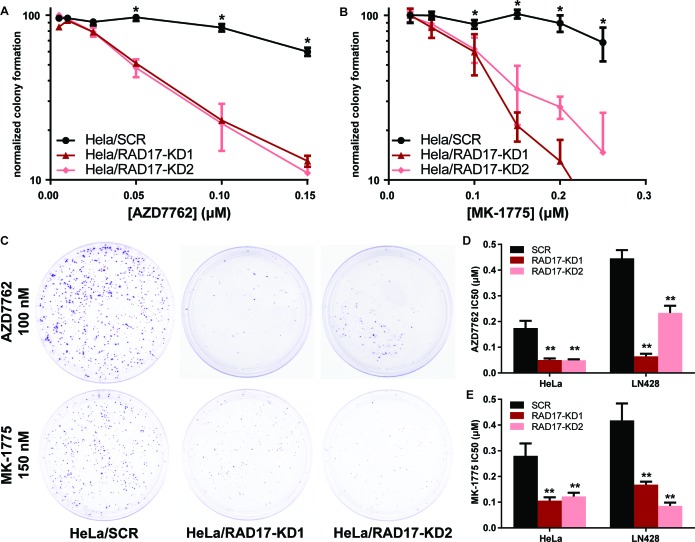
*RAD17* knockdown is synthetically lethal with CHEK1, CHEK2, and WEE1 inhibition **A., B.** HeLa cells with either stable knockdown (HeLa/RAD17-KD) or non-targeting shRNA (HeLa/SCR) were treated with either AZD7762 or MK-1775 in clonogenic assay, error bars represent +/− SD, * indicates *p* < 0.05 for *t*-test comparing SCR and RAD17-KD at that dose. **C.** Images of clonogenic plates from HeLa cells treated with either AZD7762 or MK-1775. **D., E.** IC_50_ values determined from non-linear fit of data from clonogenic experiments for AZD7762 and MK-1775 in HeLa and LN428 cells, error bars represent +/− 95% CI, ** indicates *p* < 0.0001 for extra sum-of-squares F test to comparing each RAD17-KD to SCR.

To further explore the relationship between *RAD17* and the checkpoint kinases, we created stable knockdown cell lines using lentiviral shRNA constructs targeting *RAD17* in HeLa cells, a human cancer cell line derived from a cervical adenocarcinoma, as well as LN428, a human cancer cell line derived from glioblastoma multiforme [47]. Both cell lines have inactive p53; HeLa by the effect of human papillomavirus (HPV) gene E6, and LN428 by somatic mutation. Effectiveness of gene knockdown was confirmed at the mRNA level by RT-qPCR, and at the protein level by both immunofluorescence and western blot (Supplementary Figure 2). The dual CHEK1/2 inhibitor AZD7762 (chemical structures shown in Supplementary Figure 3) was significantly more toxic to HeLa cells with *RAD17* knockdown relative to non-targeting controls (F-test *p* < 0.0001, Figure 2A and 2C, Table 1). AZD7762 was also significantly more toxic to *RAD17* knockdowns in LN428 cells (F-test *p* < 0.0001, Figure 2D, Supplementary Figure 4A). Additionally we performed 2way ANOVA to assess what portion of the difference in viability was due to *RAD17* knockdown. In HeLa cells 19.5% of the variation was from *RAD17* effect (*p* < 0.0001) and 13.7% from interaction between RAD17 effect and dose of drug (*p* < 0.0001), for LN428 these percentages were 38.6% and 4.3% respectively (*p* < 0.0001, Supplemental Table 4). The WEE1 inhibitor MK-1775 was significantly more toxic to *RAD17* knockdowns relative to non-silencing control in clonogenic assays in both HeLa and LN428 cells (F-test and ANOVA *p* < 0.0001, Figure 2B-2E, Supplementary Figure 4b).

**Table 1:** IC50 values from clonogenic assays

		IC50 (nM)			ratio relative to SCR	
Chemical	cell line	SCR	RAD17-KD1	RAD17-KD2	RAD17-KD1	RAD17-KD2
AZD7762	HeLa	180	50	50	3.5	3.5
MK-1775	HeLa	280	110	120	2.6	2.3
MK-8776	HeLa	3700	1900	1800	2.0	2.1
LY2603618	HeLa	5800	1100	710	5.1	8.2
BML-277	HeLa	4200	3000	3200	1.4	1.3
AZD-MK1775 combo	HeLa	41	14	11	2.9	3.8
AZD7762	LN428	450	65	230	6.9	1.9
MK-1775	LN428	420	170	86	2.5	4.9
AZD-MK1775 combo	LN428	97	67	31	1.5	3.1

Several structurally distinct checkpoint kinase inhibitors were tested to determine if the observed synthetic lethality was an on-target effect. *RAD17* knockdown sensitized HeLa cells to the CHEK1 selective inhibitors MK-8776 and LY2603618 (Supplementary Figure 4c and d, Table 1). The CHEK2 selective inhibitor BML-277 also showed greater toxicity to HeLa cells with *RAD17* knockdown, although the magnitude of the synthetic lethal effect was less than that seen with CHEK1 inhibitors (Supplementary Figure 4e and f). *CHEK1*, *CHEK2*, and *WEE1* were also knocked down transiently using siRNA. The cytotoxic effect of *WEE1* knockdown was significantly greater in LN428/RAD17-KD cells than controls (26.8% vs. 60.2% normalized viability, *p* = 0.017, Supplementary Figure 4g). As single knockdowns, both *CHEK1* and *CHEK2* had a mild effect on viability, trending towards *RAD17* knockdowns being more sensitive. LN428/RAD17-KD cells were significantly more sensitive to simultaneous knockdown of *CHEK1* and *CHEK2* than LN428/SCR control (50.9% vs. 89.6% normalized viability, *p* = 0.011, Supplementary Figure 4g). Overall, these results demonstrate that *RAD17* has a synthetic lethal relationship with each of the checkpoint kinases *CHEK1*, *CHEK2*, and *WEE1*.

### Combined CHEK1/2 and WEE1 inhibition causes synergistic toxicity in the setting of *RAD17* loss-of-function

**Figure 3:**
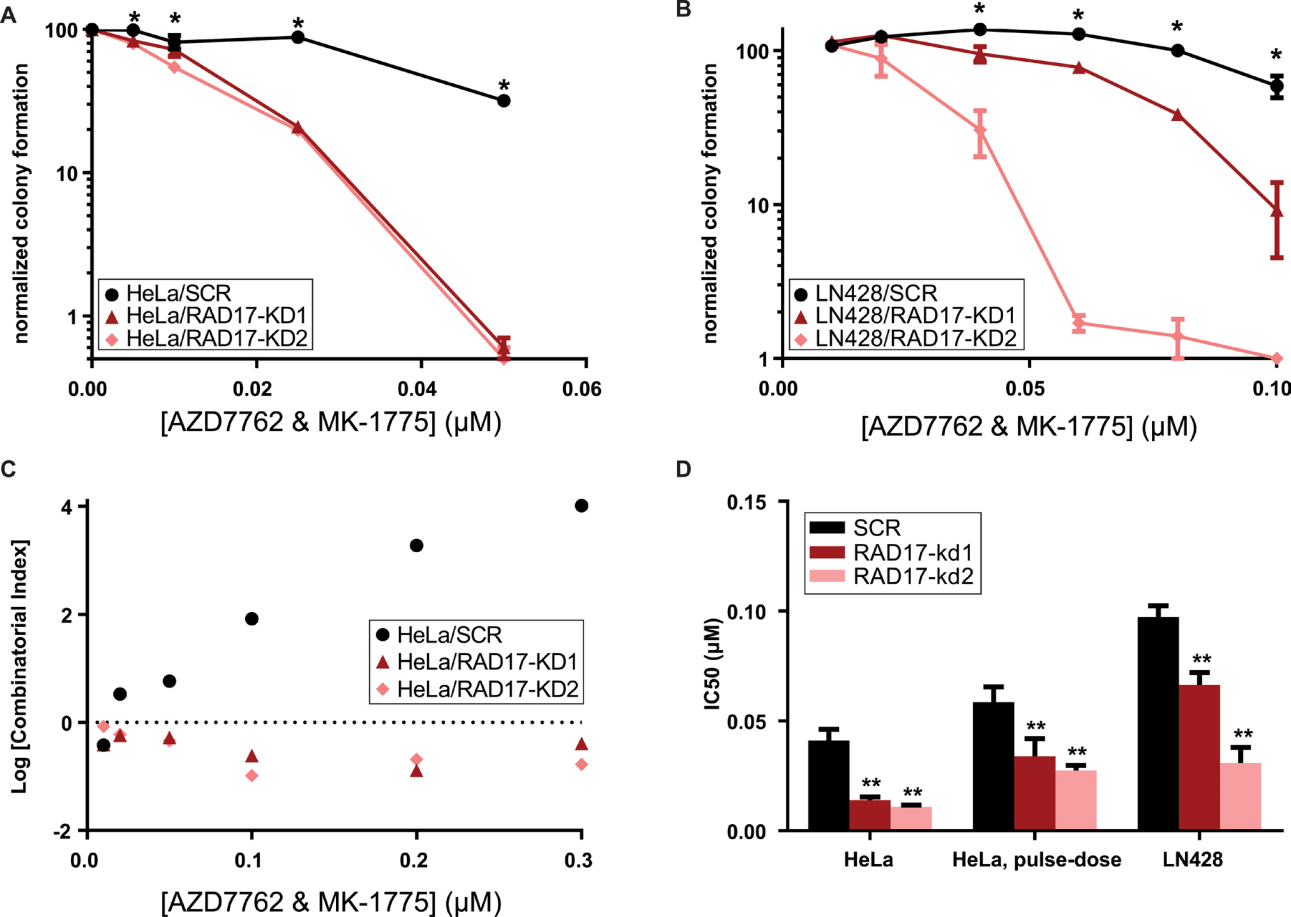
Dual inhibition with AZD7762 and MK-1775 results in synergistic toxicity in *RAD17* knockdown cell lines **A.**, **B.** Clonogenic assay combining both AZD7762 and MK-1775 in HeLa cells and LN428 cells error bars represent +/− SD, * indicates *p* < 0.05 for *t*-test comparing SCR and RAD17-KD at that dose. **C.** Log Combinatorial Index as determined by method of Chou & Talalay from HeLa clonogenic experiment, values less than zero indicate synergy, values above zero indicate antagonism. **D.** IC_50_ values determined from non-linear fit of data from clonogenic experiments, error bars represent +/− 95% CI, ** indicates *p* < 0.0001 for extra sum-of-squares F test to comparing each RAD17-KD to SCR.

Given prior reports of synergy between CHEK1 and WEE1 inhibitors in lymphoma [48], leukemia [11], and solid tumor [12, 49] cell lines, we suspected that simultaneous inhibition of CHEK1 and WEE1 might further magnify the synthetic lethal effect with *RAD17* knockdown. Indeed, the combination of AZD7762 with an equal dose of MK-1775 was more toxic to *RAD17* knockdown cells than non-silencing controls in both HeLa (F-test and ANOVA *p* < 0.0001, Figure 3a) and LN428 cells (F-test and ANOVA *p* < 0.0001, Figure 3B). The absolute IC_50_ concentration was approximately four-fold lower for the combination relative to the IC_50_ concentrations of either AZD7762 or MK-1775 alone (Table 1). However, the magnitude of the synthetic lethal effect, that is the ratio of wild type IC_50_ to *RAD17* knockdown IC_50_, was essentially the same for the combination relative to either single molecule. The combination of AZD7762 with MK-1775 was synergistic in both *RAD17* knockdown cell lines but not in non-silencing controls for all doses tested (Figure 3C). Additionally we tested the ATR inhibitor VX-970, which is currently in Phase I/II clinical testing in combination with topotecan [50]. *RAD17* knockdown sensitized both HeLa and LN428 cells to VX-970, reducing IC50 by approximately 3 fold (F-test and ANOVA *p* < 0.005, Supplemental Figure 5a and b). The combination of AZD7762 with VX-970 was also more potent in HeLa cells with *RAD17* knockdown (F-test and ANOVA *p* < 0.0001, Supplemental Figure 5c). Interestingly, the combination of AZD7762 with VX-970 was synergistic in both HeLa cells with *RAD17* knockdown as well as non-targeting controls (Supplementary Figure 5d).

Given that evidence of forced mitotic entry has been observed as soon as 8 hours after treatment with WEE1 inhibitors [13], we suspected that transient CHEK1/2-WEE1 inhibition would be sufficient to kill *RAD17* knockdown cells. A pulse-dose exposure of AZD7762 with MK-1775 for 72 hours was only slightly less toxic than continuous exposure and again demonstrated a synthetic lethal effect with *RAD17* knockdown (Figure 3D). This suggests that intermittent dosing of CHEK1 or WEE1 inhibitors could be a viable therapeutic strategy in certain susceptible tumors.

### *RAD17* synthetic lethal effect with checkpoint kinases is associated with increase in γH2AX accumulation

**Figure 4:**
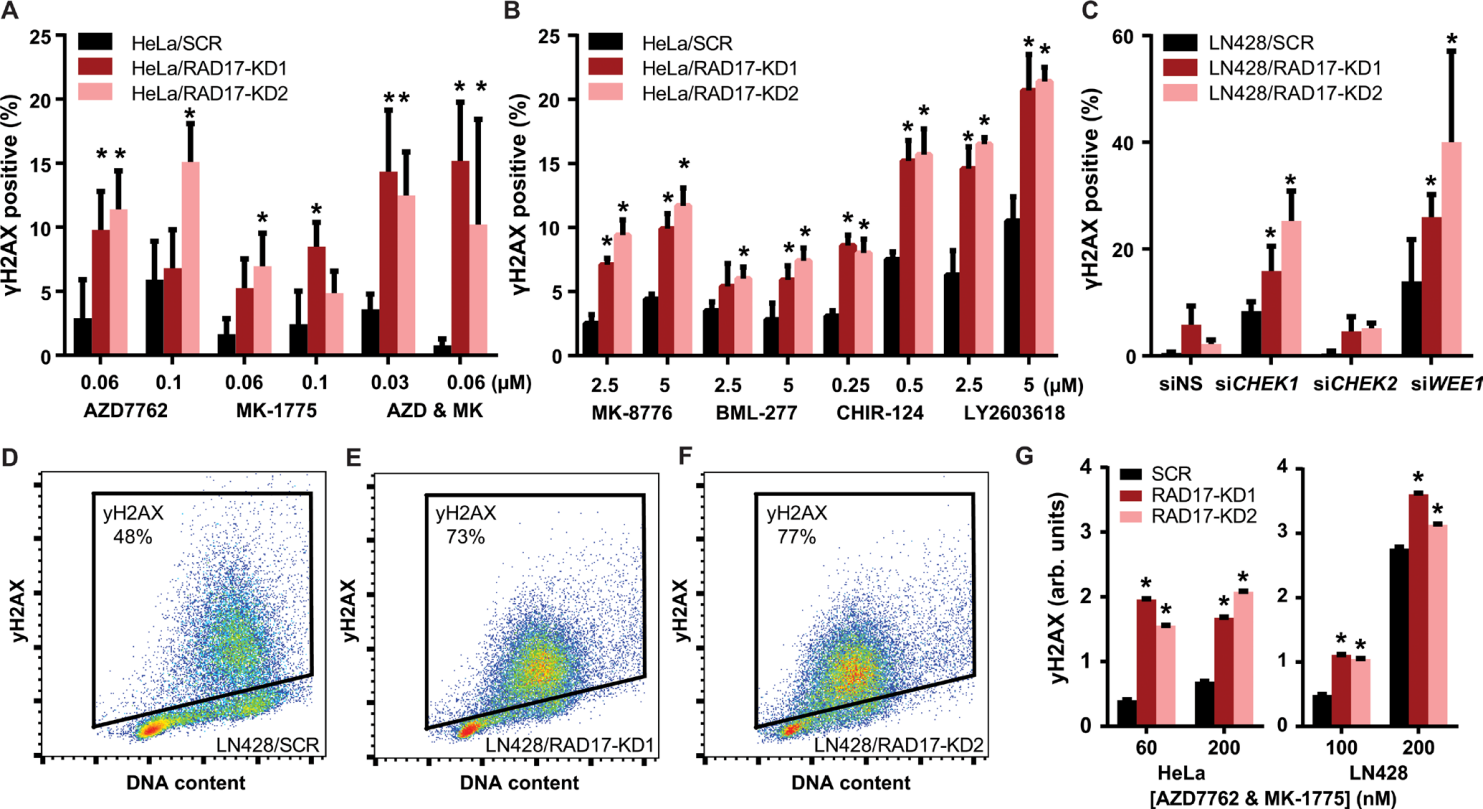
*RAD17* knockdown exacerbates accumulation of DNA damage following checkpoint kinase inhibition **A.** Percentage of HeLa cells staining positive for γH2AX by immunofluorescence when treated with AZD7762, MK-1775, or the combination of both, error bars represent +/− SD, * indicates *p* < 0.05 for *t*-test comparing each RAD17-KD to SCR at that dose. **B.** Similar experiment to **A.** except with MK-8776, BML-277, CHIR-124, and LY2603618. **C.** Percentage of LN428 cells staining positive for H2AX by immunofluorescence when treated with non-silencing siRNA (siNS) or siRNA targeting *CHEK1*, *CHEK2*, or *WEE1*, error bars represent +/− SD, * indicates *p* < 0.05 for *t*-test comparing each RAD17-KD to SCR at that dose. Scatter plots showing gating for H2AX positive cells for LN428/SCR **D.**, LN428/RAD17-KD1 **E.**, or LN428/RAD17-KD2 **F.** for samples treated with combination of AZD7762 and MK-1775 both at 200 nM. **G.** Bar graph showing average γH2AX intensity by FACS for population of HeLa or LN428 cells treated at given doses, error bars represent +/− 95% CI, * indicates *p* < 0.05 for *t*-test comparing each RAD17-KD to SCR at that dose.

*RAD17* is known to participate in cell cycle regulation by activating the S-phase checkpoint [51] in addition to its role in DNA damage repair [34]. We sought to determine which function was mediating the observed synthetic lethal effect with checkpoint kinase inhibition using a high-throughput immunofluorescence assay to measure phosphorylation of histone H2AX at Ser139 (γH2AX), an established marker of DNA damage [52]. It has previously been reported that inhibition of CHEK1 or WEE1 causes accumulation of γH2AX [2, 7, 12]. We found that chemical inhibition of CHEK1/2 with AZD7762 resulted in a dose dependent increase in γH2AX, with significantly greater induction of γH2AX seen in *RAD17* knockdown samples relative to non-silencing controls in both HeLa (*p* < 0.05, Figure 4A) and LN428 cell lines (*p* < 0.05, Supplementary Figure 6a). Similarly, WEE1 inhibition with MK-1775 also resulted in a dose dependent increase in γH2AX with significantly greater induction of γH2AX seen in *RAD17* knockdown samples in both HeLa and LN428 cell lines (*p* < 0.05, Figure 4A and Supplementary Figure 6a). Similar to its effect in clonogenic assay, the combination of both AZD7762 and MK-1775 was more potent than either single agent in terms of induction of γH2AX. The increase in γH2AX seen with combined treatment was greater in the setting of *RAD17* knockdown (*p* < 0.05, Figure 4A and Supplementary Figure 6a). The CHEK1 selective inhibitors MK-8776, CHIR-124, and LY2603618 also increased γH2AX accumulation to a greater degree in cells with *RAD17* knockdown (*p* < 0.05, Figure 4B). The CHEK2 selective inhibitor BML-277 induced less γH2AX than the CHEK1 inhibitors, but more γH2AX was observed in *RAD17* knockdown cells relative to non-silencing controls (*p* < 0.05, Figure 4B). Transient knockdown of *CHEK1*, *CHEK2*, and *WEE1* produced results similar to chemical inhibitors. Knockdown of either *CHEK1* or *WEE1* led to significantly more γH2AX accumulation in *RAD17* knockdown cells (*p* < 0.05, Figure 4C), however this effect was not seen with knockdown of *CHEK2*. These results confirm that the increase in γH2AX accumulation seen with AZD7762 and MK-1775 is due to the on-target effect of inhibition of CHEK1/2 or WEE1, respectively, and suggest the effect of AZD7762 is mediated primarily through inhibition of CHEK1.

DNA damage induction in response to checkpoint kinase inhibition was also assessed in a Fluorescence Activated Cell Sorting (FACS) assay. The addition of the combination of AZD7762 and MK-1775 resulted in greater accumulation of γH2AX in *RAD17* knockdown cells, consistent with the results of the immunofluorescence assay (Figure 4D-4G, Supplementary Figure 6b-d). The observation that loss-of-function of *RAD17* exacerbates the γH2AX accumulation seen with CHEK1 or WEE1 inhibition suggests that RAD17's role in DNA repair is at least partially independent of the checkpoint kinases.

**Figure 5:**
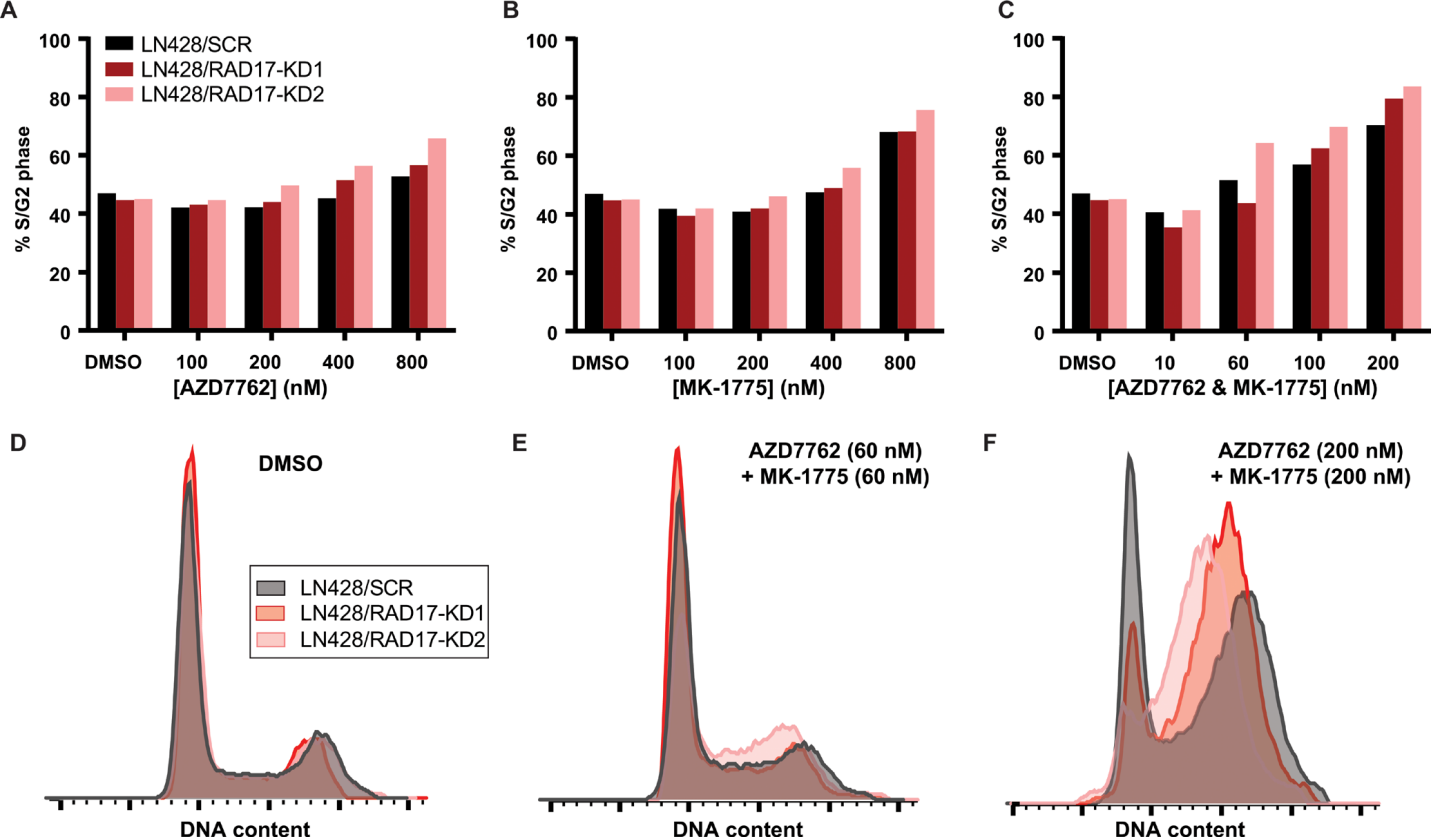
*RAD17* knockdown has minimal impact cell cycle regulation **A**.-**C**. Bar graphs summarizing percentage of LN428 cells in either S or G2 phase when treated with either AZD7762, MK-1775, or both in combination at indicated doses. **D**.-**E**. Overlaid histograms of events by DNA content showing cell cycle distributions for LN428 cells treated with DMSO, AZD7762 and MK-1775 at 60 nM, or AZD7762 and MK-1775 at 200 nM.

To evaluate the effect of *RAD17* knockdown on cell cycle progression we performed FACS. In contrast to its effect on γH2AX accumulation, *RAD17* knockdown had minimal effect on progression through the G1-S or G2/M checkpoints. In LN428 and HeLa cells a dose-dependent accumulation of cells in the S or G2 phase was seen with AZD7762, MK-1775, and the combination of AZD7762 and MK-1775 (Figure 5A-5C, Supplementary Figure 6e-g). However, the accumulation of cells in S or G2 phase was not increased by the knockdown of *RAD17* in the absence of checkpoint kinase inhibition (Figure 5D), or at doses near the IC_50_ concentration for these compounds (Figure 5E). At a dose of 200 nM, more than three times the IC_50_ for LN428/RAD17-KD1 cells or six times the IC_50_ for LN428/RAD17-KD2 cells, there was a greater percentage of cells in S or G2 phase in the *RAD17* knockdown cell lines relative to non-silencing control (Figure 5F). Similar results were seen in HeLa cells (Supplementary Figure 6h-j). These data suggest that in the setting of *RAD17* knockdown cells continue to cycle normally. The fact that cytotoxic doses of AZD7762 and MK-1775 do not cause accumulation of cells in S or G2 phase with or without *RAD17* indicates that loss of cell cycle regulation is not the primary mechanism causing cell death. This result is consistent with prior reports that WEE1 or CHEK1 inhibition can ultimately cause cell death by mitotic catastrophe [53, 54].

### Interaction of *RAD17* and checkpoint kinases in primary human tumor samples

**Figure 6:**
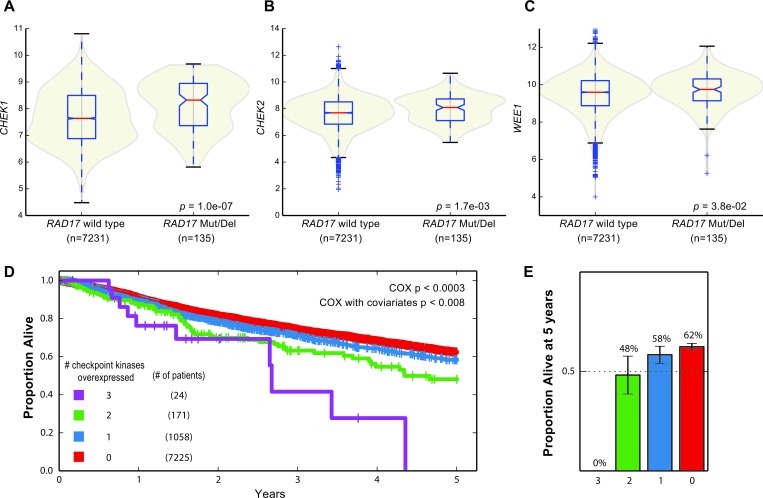
Synthetic lethal interactions with checkpoint kinases in human tumor samples **A**.-**C**.*CHEK1*, *CHEK2*, and *WEE1* are all over expressed in tumors with either homozygous deletion or mutation of *RAD17*. **D.** Kaplan-Meier plot of patients from TCGA stratified by overexpression of *CHEK1*, *CHEK2*, or *WEE1*. Red curve - patients with overexpression of none of *CHEK1*, *CHEK2*, or *WEE1*; blue curve - patients with overexpression of one of the three; green curve - overexpression of two of three; purple curve overexpression of all three. **E.** Proportion of patients alive at five years for same populations, error bars represent +/− 95% CI.

It has been demonstrated that co-disruption of synthetic lethal partners in a tumor is associated with better patient survival [55], presumably because these tumors are less robust to perturbations and thus more vulnerable to therapy. To assess whether there is evidence of interaction between *RAD17* and the checkpoint kinases in primary human tumors, we examined somatic mutation, copy number variation, and mRNA expression data from ~8000 biopsy specimens spanning multiple cancer types in TCGA. Tumors with *RAD17* homozygous deletion or mutation had significantly increased expression of both *CHEK1* and *CHEK2* relative to tumors without *RAD17* alteration (Mann-Whitney U test *p* = 1.0e-7 and 0.0017, respectively), with *WEE1* there was a non-significant trend toward increased expression (Figure 6A-6C). Given the overlapping roles of *RAD17* and the checkpoint kinases in repairing DNA damage we suspected that the observed overexpression of *CHEK1*, *CHEK2* and *WEE1* was a compensatory mechanism to prevent excessive DNA damage. To evaluate if this overexpression of checkpoint kinases is potentially clinically relevant, we next looked for an association with patient survival. At an overexpression cutoff of two sigma, the majority of TCGA patients had none of the three checkpoint kinases (*CHEK1*, *CHEK2* and *WEE1*) overexpressed. These patients had the best overall survival, and as the number of overexpressed checkpoint kinases increased, overall survival became progressively worse (Figure 6D). The number of checkpoint kinases overexpressed was significantly associated with survival as assessed by a Cox proportional hazards model (*p* < 0.003 without covariates, *p* < 0.008 with covariates age, stage, tumor type). The proportion of patients alive at five years ranged from 62% for those with no checkpoint kinase overexpression to 0% for the 24 patients overexpressing all three checkpoint kinases (Figure 6E). These results suggest that the synthetic lethal effect observed between *RAD17* and the checkpoint kinases *in vitro* may be functionally relevant *in vivo.*

## DISCUSSION

Small molecule inhibitors of either CHEK1 or WEE1 remain in clinical development, both as single agents and in combination with either DNA damaging or anti-metabolite chemotherapy [2, 4, 7]. Currently, these early phase trials are being performed without biomarker stratification due in part to a poor understanding of the molecular predictors of response to these therapies. Prior *in vitro* testing of CHEK1 inhibitors has found that only 10-15% of cancer cell lines are sensitive to isolated CHEK1 inhibition [56]. Assuming these cell lines are a reasonable surrogate for human tumors, it suggests that for each patient with a tumor sensitive to checkpoint kinase inhibition, as many as nine patients with resistant tumors will be treated with ineffective therapy. To address this need we identified several TSG involved in either DNA repair or cell cycle regulation to be synthetically lethal with the checkpoint kinase inhibitor AZD7762. Focusing on *RAD17*, we show in clonogenic assay that shRNA mediated knockdown of *RAD17* increases the sensitivity of either HeLa or LN428 cancer cells to both chemical inhibition or siRNA mediated knockdown of the checkpoint kinases *CHEK1*, *CHEK2* and *WEE1*. Evidence of the interaction between *RAD17* and *CHEK1/2* was also seen in a functional genomic screen involving a panel of over 100 cell lines and in the budding yeast *S. cerevisiae*.

The presence of a strong conserved genetic interaction between *RAD17* and the checkpoint kinases in human and yeast species separated by up to a billion years of evolution [22], suggests that this functional relationship is not just active in some conditions or cell states but may be fundamental for eukaryotic life. This supposition is supported by the fact that (excluding hyper-mutated tumors) there are no occurrences of tumors with mutations in both *RAD17* and either *CHEK1*, *CHEK2* and *WEE1* across all cancer types in TCGA. We suspect that the observed overexpression of *CHEK1*, *CHEK2* and *WEE1* in tumors with *RAD17* deletion or mutation is a compensatory response to impaired DNA damage repair. Tumors that overexpress checkpoint kinases should have greater fitness than those without compensatory overexpression, resulting in worse clinical outcomes for these patients, as we observed.

Synthetic lethal interactions are predicted to occur between genes that participate in independent, but complementary pathways, such as base excision repair and HR for the synthetic lethal pair *PARP1* and *BRCA1* [22]. Given that *CHEK1*, *CHEK2,* and *WEE1* play a role in the repair of DNA damage in addition to regulating cell cycle checkpoints [2, 18, 57], we suspected that the interaction with *RAD17* would involve one or both of these two functions. Our results suggest that it the role of *RAD17* in DNA damage repair, likely the recruitment of the MRN complex to DSB, which becomes essential in the setting of checkpoint kinase inhibition. This conclusion is supported by prior data in HeLa cells identifying that claspin-dependent activation of CHEK1 is independent of *RAD17* [58].

Although the compound AZD7762, which inhibits both CHEK1 and CHEK2, is no longer in clinical development due to cardiac toxicity [9], other selective inhibitors of CHEK1 including MK-8776 remain in clinical development. It is unknown if the cardiac issues seen with AZD7762 relate to dual CHEK1/2 inhibition or an off-target effect; regardless, our data on the selective CHEK1 inhibitors MK-8776, LY2603618, and CHIR-124 suggest that CHEK1 inhibition is sufficient to achieve a synthetic lethal interaction with *RAD17* loss-of-function. The selective CHEK1 inhibitors were not tried in combination with WEE1 inhibition in this study, but given a prior report of synergy between MK-8776 and MK-1775 in the majority of a set of 39 cancer cell lines [12], it is likely that a CHEK1 selective inhibitor would perform similarly to AZD7762 when combined with MK-1775 in the setting of *RAD17* loss-of-function. The combination of CHEK1 inhibitor and WEE1 inhibitor shows particular promise in *RAD17* mutant or deleted tumors, as the IC_50_ of these drugs in combination is four-fold lower than that of each drug individually. The fact that only a pulse-dose of CHEK1 and WEE1 inhibition was needed to achieve a synthetic lethal effect in *RAD17* knockdown cells suggests the possibility that this combination could be used as a long term maintenance therapy, free of traditional cytotoxic chemotherapy.

## MATERIALS AND METHODS

### Chemo-genetic screen

A dose-response curve for AZD7762 in HeLa cells was created prior to screening. HeLa cells were seeded at density of 500 cells per well in 384 well plates, after 72 hours of drug exposure viability was measured using the Cell Titer Glow (Promega) viability regent. Prism v6.05 (GraphPad Software) was used to fit non-linear regression to create a dose-response curve which determined IC_20_ (0.22 μM) and IC_40_ (0.4 μM) doses. For the chemo-genetic screen cells were transfected by wet reverse method using Lipofectamine (Life Technologies). Each gene was targeted by four individual siRNA constructs pooled in the same well; three replicates were performed on separate plates for each dose. Correlation of replicates was 0.97 indicating excellent reproducibility (Figure S1B-D). Synthetic lethal interactions were scored by first normalizing for the viability effect of gene knockdown in the presence of only dimethyl-sulfoxide (DMSO) solvent, then comparing these normalized values for each gene to panel of non-silencing controls to determine Z-score. Since the Z-scores for the IC_20_ and IC_40_ doses were highly correlated (r = 0.87, *p* < 0.0001, Figure S1A) they were averaged to create a single value for each of the 112 genes screened.

### cBioPortal analysis

Data from all available cohorts on cBioPortal (www.cbioportal.org) excluding cell lines was last downloaded on 6/1/15.

### Sequence alignment

The online version of Clustal W, version 2.1 was used to perform sequence alignment.

### Yeast spot dilution and synthetic genetic array assay

Yeast mutant strains were constructed by the pinning robot ROTOR (Singer Instruments) using SGA technology [59]. Colony sizes were quantified and normalized using Colony Analyzer to assess viability [60]. For spot dilution assays, cells were grown to mid-log in rich media (YPAD). Aliquots of 10-fold serial dilutions were spotted on rich media (YPAD) and grown for 2 days at 30°C.

### Project achilles analysis

Raw shRNA viability data was downloaded from Cheung *et al*, 2011 [45]. Viability for each gene was determined by averaging the values of five independent constructs.

### Generation of lentiviral knockdown cell lines

The shuttle vectors for expression of shRNA targeting each gene were purchased from Sigma (St. Louis, MO). Lentiviruses were prepared in collaboration with the UPCI Lentiviral facility. Lentiviral particles were generated by co-transfection of 4 plasmids (the shuttle vector plus three packaging plasmids: pMD2.g (VSVG), pVSV-REV and PMDLg/pRRE) into 293-FT cells using FuGene 6 Transfection Reagent (Roche). 10,000 cells were seeded into a 6-well plate 24 hours before transduction. Cells were transduced for 18 hours at 32°C and then cultured for 8 hours at 37°C. Next, the cells were transduced a second time at 32°C for 18 hours with the lentiviruses containing the same shRNA, and then cultured for 24 hours at 37°C. Cells were selected by culturing in growth media with 1.0 μg/mL puromycin for two weeks to obtain stable knockdown cells. For each gene, five individual shRNAs targeting each gene were used to generate five independent knockdown cell lines. The cell lines with the highest level of knockdown were selected for future studies.

### Determination of gene knockdown level (RT-qPCR)

Gene expression (mRNA) was measured by quantitative reverse transcription-PCR (qRT-PCR) using an Applied Biosystems StepOnePlus system. Briefly, 80,000 cells were lysed and reverse transcribed using the Taqman Gene Expression Cells-to-CT kit (Applied Biosystems). Each sample was analyzed in duplicate using a Taqman Gene Expression Assay for human *RAD17* (Hs00607830_m1) and normalized to the expression of human β-actin (Applied Biosystems). Expression (mRNA) was analyzed via the ΔΔCT method, results are reported as an average of two analyses +/− SE.

### Determination of gene knockdown level (Immunofluorescence and western blot)

Cells were seeded into clear bottom 384 well plates (Nunc), fixed with 4% formaldehyde, blocked with 2% bovine serum albumin in TBST, and stained with Hoechst and anti-RAD17 (Abnova) primary antibody followed by Alexa594 donkey anti-mouse secondary antibody. Plates were imaged with ImageXpress Micro automated epi-fluorescent microscope (Molecular Devices) and images were scored with MetaExpress analysis software (Molecular Devices). For western blot cells were lysed with RIPA buffer and prepared for SDS-PAGE using NuPAGE kit (Invitrogen). Same anti-RAD17 (Abnova) primary antibody was used as in immunofluorescence assays.

### Clonogenic assays

Cells were counted using Scepter automated cell counter (Millipore) and between 800-2000 cells were seeded per plate. Cells were treated with small molecule inhibitors or DMSO solvent control for 9 days (HeLa) or 10 days (LN428). Consistent with standard protocol a cut off of 50 cell was used as threshold to define a colony [61]. Canon Rebel T3i digital camera was used to create a digital image of each plate, colonies were then scored using a custom Matlab script calibrated against manually counted control plates for each cell line. Number of colonies per plates was normalized to number of colonies on plates treated only with DMSO solvent, each lentiviral modified cell line was normalized independently. IC_50_ concentrations were determined by performing four parameter non-linear regression using Prism v6.05 software. IC_50_ concentrations compared to each other using extra sum-of-squares F test. Pulse-dose experiments were performed by exchanging media to remove drugs after 72 hours of exposure with colony formation measured after an additional seven additional days of growth. The method of Chou and Talalay was used to measure synergistic effects of drug combination [62].

### γH2AX immunofluorescence assay

250-500 cells were seeded into clear bottom 384 well plates (Nunc) and treated with either siRNA, kinase inhibitors, or controls. After incubation with either siRNA or small molecule inhibitor for 48-72 hours cells were fixed with 4% formaldehyde, blocked with 2% bovine serum albumin in TBST, and stained with Hoechst and FITC conjugated anti- γ-H2AX antibody (Millipore). Plates were imaged with ImageXpress Micro automated epi-fluorescent microscope (Molecular Devices) and images were scored with MetaExpress analysis software (Molecular Devices). At baseline without any pharmacological or genetic intervention, there was a non-significant trend towards more cells scoring positive for γH2AX in RAD17-KD cell lines relative to non-silencing control.

### FACS assay

400,000 - 500,000 cells were plated in 10 cm dishes and allow to attach overnight before being treated with small molecules the next day. After 48 hrs of drug exposure cells were harvested by incubating with trypsin for 5 min. Trypsin was neutralized with serum containing media and then cells were pelleted and re-suspended in ice cold 70% ETOH and stored at −20 C. On day of FACS run cells were washed once with PBS and incubated with FITC conjugated anti- γ-H2AX antibody (Millipore) per manufacturer protocol. Cells were then suspended in DNA staining buffer (Sodium citrate 0.1%, Triton-X 100 0.3%, propridium iodide 0.1 mg/mL, ribonuclease A 0.2 mg/mL in distilled water) and run on FACS machine (B&D LSRII, BD Biosciences). FACS data was analyzed with FlowJo v10.0.8 (Tree Star, Inc). Cell cycle analysis was performed using the Watson (univariate) method with constraint of equal CV for 2N and 4N peaks [63]. At baseline, approximately 40% of cells were in S or G2 phase for both RAD17-KD and non-silencing cell lines in both the HeLa and LN428 background.

### TCGA analysis

Data for TCGA cohort were obtained from the Genome Data Analysis Center (GDAC) Firehose website, latest data were downloaded from the 4/2/15 standard data and analyses run.

## SUPPLEMENTARY MATERIAL FIGURES AND TABLES




