# Supplementary material for: Chemogenetic profiling identifies RAD17 as synthetically lethal with checkpoint kinase inhibition
Source: Oncotarget. 2015 Sep 30;6(34):35755–69. doi: 10.18632/oncotarget.5928 (PMC4742139; doi:10.18632/oncotarget.5928)
Supplement: Supplementary file 1 [file oncotarget-06-35755-s001.pdf]

Chemogenetic profiling identifies *RAD17* as synthetically lethal with checkpoint kinase inhibition

Supplementary Material

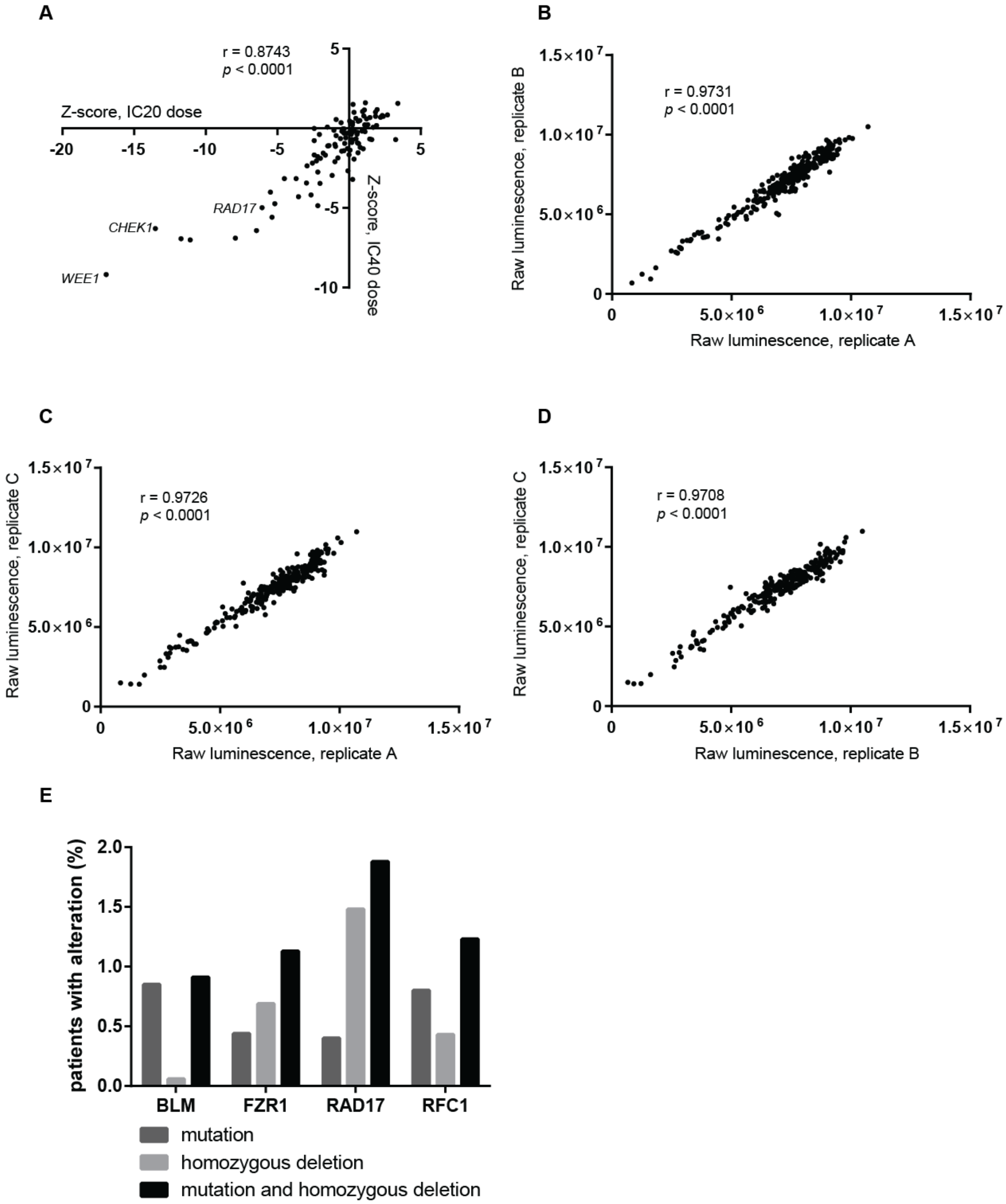

Supplementary Figure 1. Quality control metrics, chemo-genetic profiling of AZD7762. (**A**) Pearson correlation of Z-scores for each gene for IC_{20} vs. IC_{40} dose of AZD7762, each point represents one gene. (**C-D**) Pairwise Pearson correlation of raw luminescence values for A, B, and C replicates, each point represents one well of a 384 well plate. (**E**) Frequency of mutation or homozygous deletion of the genes *BLM, FZR1, RAD17 and RFC1.*

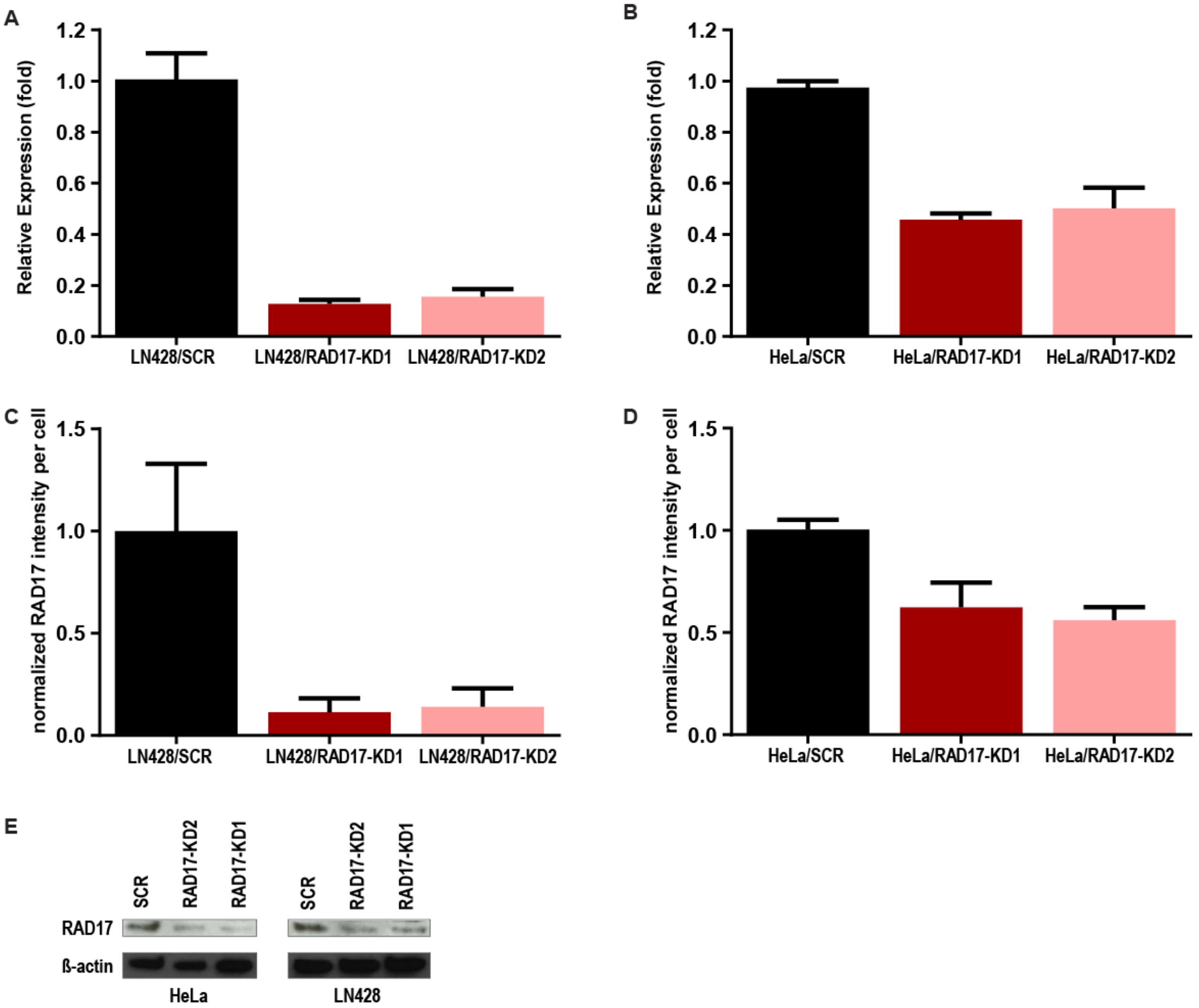

Supplementary Figure 2. Validation shRAD17 knockdown cell lines. (**A**) qPCR of *RAD17* mRNA in LN428 cell lines, error bars represents +/- SEM. (**B**) qPCR of *RAD17* mRNA in HeLa cell lines, error bars represents +/- SEM. (**C**) Quantitation of RAD17 protein by immunofluorescence with anti-RAD17 antibody in LN428 cells, error bars represents +/- SEM. (**D**) Quantitation of RAD17 protein by immunofluorescence with anti-RAD17 antibody in HeLA cells, error bars represents +/- SEM. (**E**) Western blot showing knockdown of RAD17 relative to β-actin control.

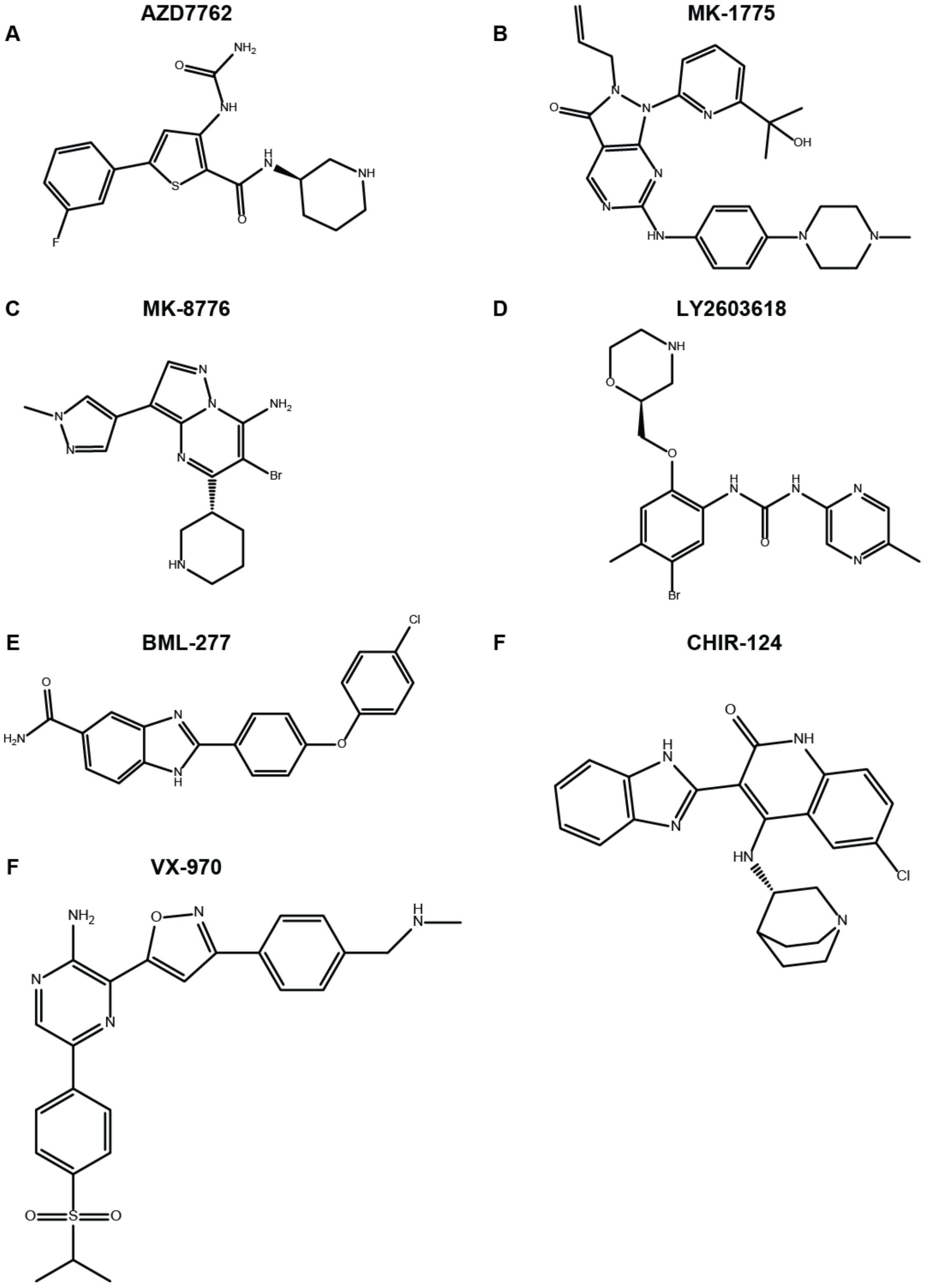

Supplementary Figure 3. Chemical structures of checkpoint kinase inhibitors. (**A**) AZD7762, (**B**) MK-1775, (**C**) MK-8776, (**D**) LY2603618, (**E**) BML-277, (**F**) CHIR-124, (**G**) VX-970.

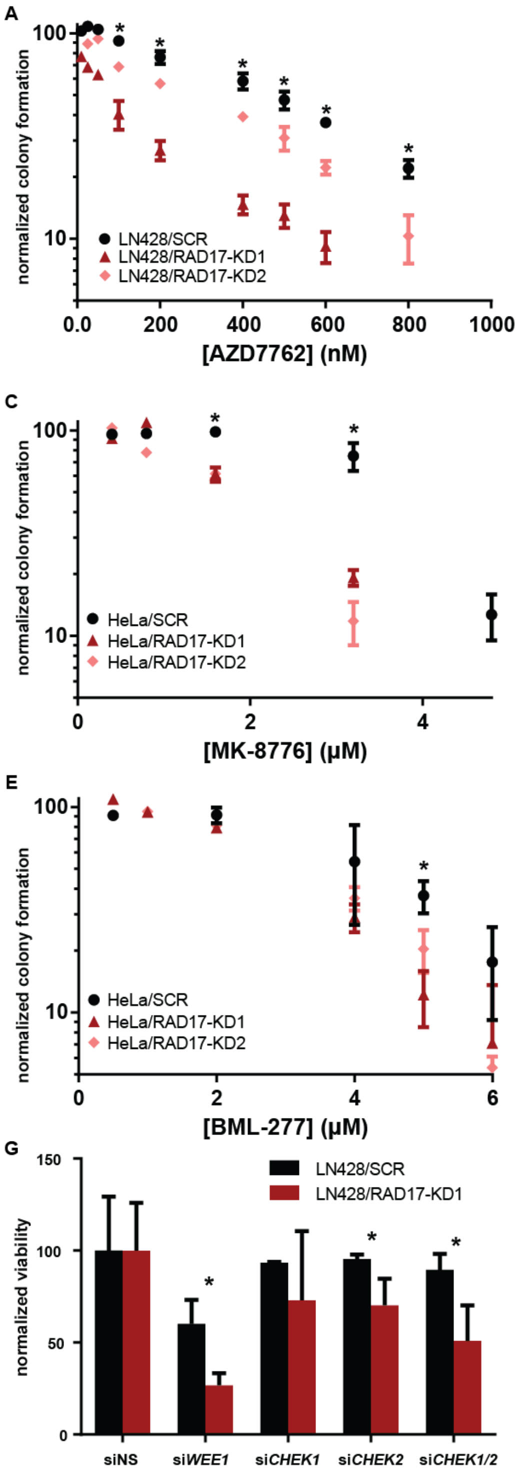

Supplementary Figure 4. *RAD17* knockdown is synthetically lethal with CHEK1, CHEK2, and WEE1 inhibition. LN428 or HeLa cells with either stable *RAD17* knockdown or non-targeting shRNA (SCR) were treated with AZD7762 (**A**), MK-1775 (**B**), MK-8776 (**C**), LY2603618 (**D**), or BML-277 (**E**) in clonogenic assay, error bars represent +/- SD, *****indicates that $p < 0.05$ for t-test between SCR and RAD17-KD at that dose. (**F**) IC_{50} values determined from non-linear fit of data from clonogenic experiments for MK-8776,

LY2603618, and BML-277 in HeLa cells, error bars represent +/- 95% CI, ** indicates $p < 0.0001$ for extra sum-of-squares F test comparing each RAD17-KD to SCR. (**G**) Viability of LN428/RAD17-KD or LN428/SCR cells transfected with indicated siRNA. Viability normalized to non-silencing control, error bars represent +/- SD, * indicates $p < 0.05$ for t-test comparing RAD17-KD to SCR.

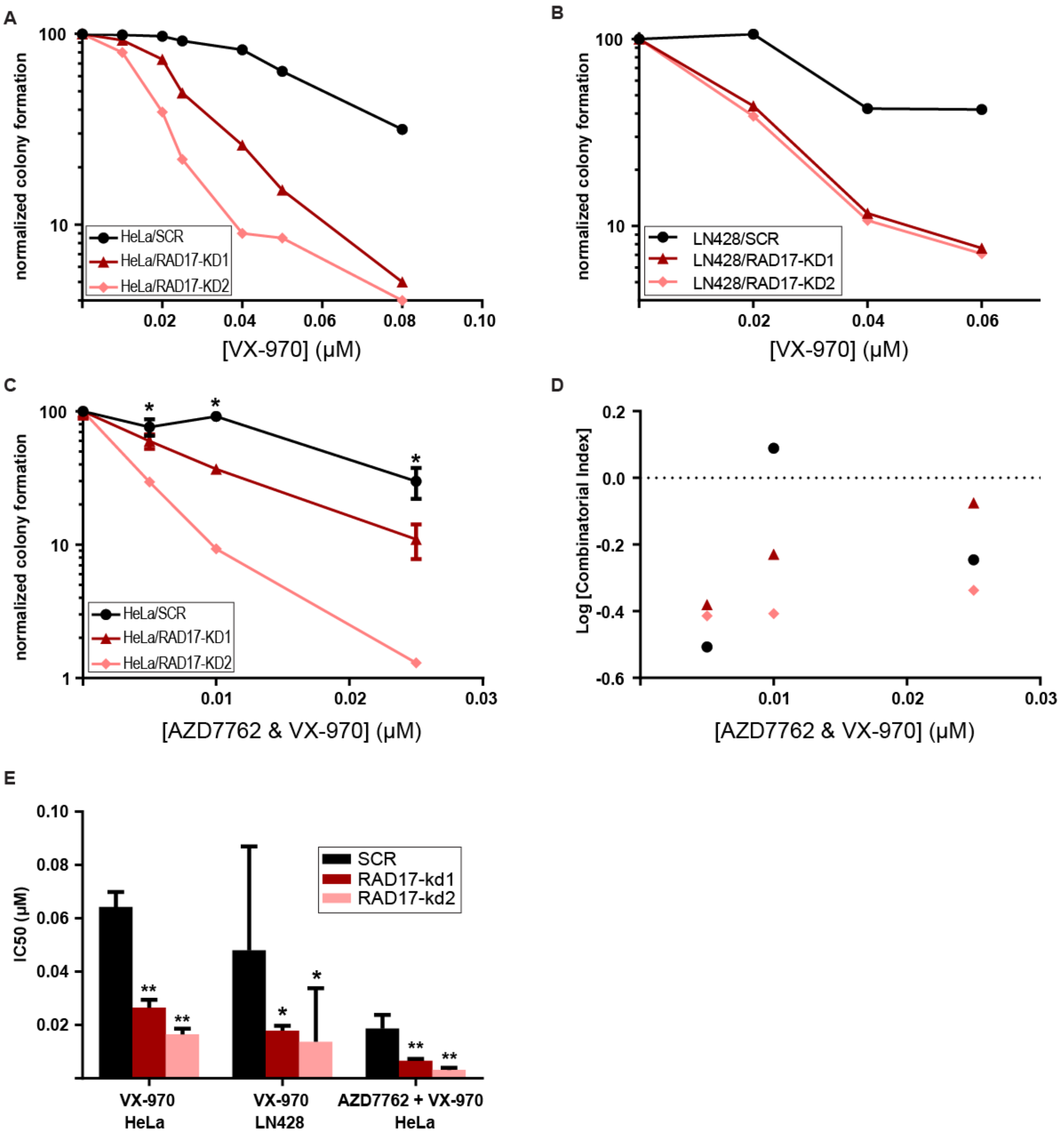

Supplementary Figure 5. *RAD17* knockdown is synthetically lethal with ATR inhibition. HeLa cells **(A)** or LN428 cells **(B)** with either stable *RAD17* knockdown or non-targeting shRNA (SCR) were treated with VX-970 in clonogenic assay. **(C)** HeLa cells were treated with the combination of AZD7762 and VX-970 in clonogenic assay, error bars represent +/- SD, *****indicates that $p < 0.05$ for t-test between SCR and RAD17-KD at that dose. **(D)** Log Combinatorial Index as determined by method of Chou & Talalay from HeLa clonogenic experiment, values less than zero indicate synergy, values above zero indicate antagonism. **(E)** IC_{50} values determined from non-linear fit of data from clonogenic experiments for VX-970 and combination of VX-970 and AZD7762, error bars represent +/- 95% CI, ****** indicates $p < 0.001$ for extra sum-of-squares F test to comparing each RAD17-KD to SCR, ***** indicates $p < 0.01$.

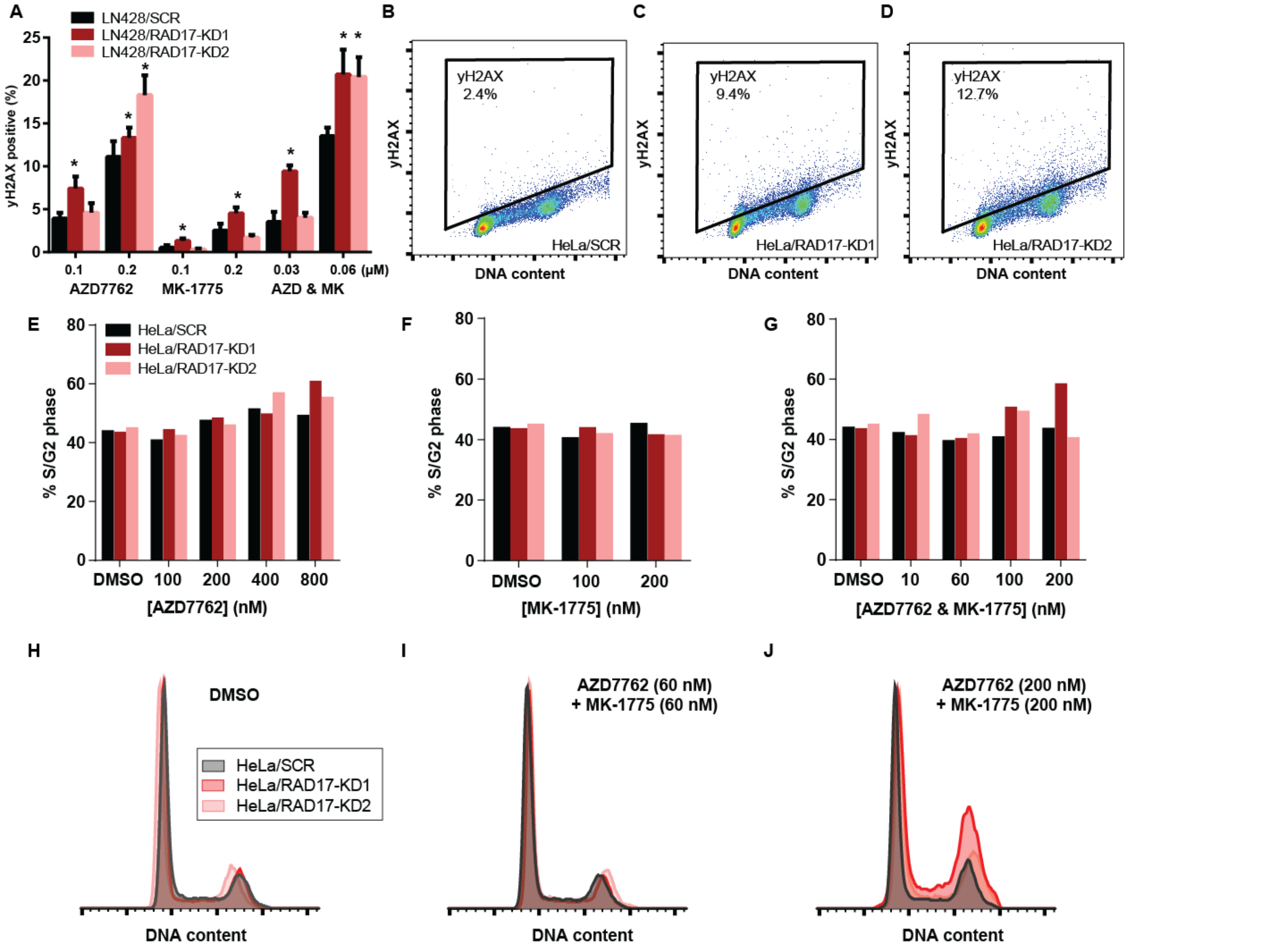

Supplementary Figure 6. DNA damage and cell cycle regulation in setting of *RAD17* knockdown. **(A)** Percentage of LN428 cells staining positive for γH2AX by immunofluorescence when treated with AZD7762, MK-1775, or the combination of both**,** error bars represent +/- SD, ***** indicates $p < 0.05$ for t-test comparing each RAD17-KD to SCR at that dose. Scatter plots showing gating for γH2AX positive cells for HeLa/SCR **(B),** HeLa/RAD17-KD1 **(C),** or HeLa/RAD17-KD2 **(D)** cells treated with combination of AZD7762 and MK-1775 both at 60 nM. Bar graphs summarizing percentage of HeLa cells in either S or G2 phase when treated with either AZD7762 **(E)**, MK-1775 **(F),** or both in combination **(G)** at indicated doses. Overlaid histograms of events by DNA content showing cell cycle distributions for HeLa cells treated with DMSO **(H),** AZD7762 and MK-1775 at 60 nM **(I)**, or AZD7762 and MK-1775 at 200 nM **(J).**

Supplemental Table 1: Z-scores for all 112 genes tested in AZD7762 chemo-genetic screen

Only Hits

Z-Score	Symbol	Entrez ID	Gene Name
-13.07	WEE1	7465	WEE1 G2 checkpoint kinase
-9.90	CHEK1	1111	checkpoint kinase 1
-9.33	CDC6	990	cell division cycle 6
-9.04	CDC73	79577	cell division cycle 73
-7.42	BLM	641	Bloom syndrome, RecQ helicase-like
-6.44	RFC1	5981	replication factor C (activator 1) 1
-5.53	RAD17	5884	RAD17 homolog (S. pombe)
-5.48	FZR1	51343	fizzy/cell division cycle 20 related 1

All Genes

Z-Score	Symbol	Entrez ID	Gene Name
0.67	ANK1	286	ankyrin 1, erythrocytic
-0.89	APC	324	adenomatous polyposis coli
0.09	ARID1A	8289	AT rich interactive domain 1A (SWI-like)
-4.96	ATAD5	79915	ATPase family, AAA domain containing 5
-0.01	ATM	472	ataxia telangiectasia mutated
-3.43	ATR	545	ATR serine/threonine kinase
-1.23	BAX	581	BCL2-associated X protein
-0.23	BECN1	8678	beclin 1, autophagy related
-7.42	BLM	641	Bloom syndrome, RecQ helicase-like
-0.19	BRCA1	672	breast cancer 1, early onset
-1.30	BRCA2	675	breast cancer 2, early onset
-0.46	BRD3	8019	bromodomain containing 3
-3.20	BRD4	23476	bromodomain containing 4
0.87	CALCR	799	calcitonin receptor
0.66	CASP3	836	caspase 3, apoptosis-related cysteine peptidase
2.48	CCNC	892	cyclin C
-3.91	CDC14A	8556	cell division cycle 14A
0.89	CDC25A	993	cell division cycle 25A
0.55	CDC25B	994	cell division cycle 25B
-0.72	CDC25C	995	cell division cycle 25C
-9.33	CDC6	990	cell division cycle 6
-9.04	CDC73	79577	cell division cycle 73
0.90	CDK1	983	cyclin-dependent kinase

-1.24	CDK12	51755	cyclin-dependent kinase 12
0.21	CDK2	1017	cyclin-dependent kinase 2
-0.42	CDKN1B	1027	cyclin-dependent kinase inhibitor 1B
-1.94	CDKN2A	1029	cyclin-dependent kinase inhibitor 2A
-9.90	CHEK1	1111	checkpoint kinase 1
1.11	CHEK2	11200	checkpoint kinase 2
1.43	CSNK1D	1453	casein kinase 1, delta
-3.42	DAAM1	23002	dishevelled associated activator of morphogenesis 1
-0.83	DDX11	1663	DEAD/H (Asp-Glu-Ala-Asp/His) box helicase 11
1.56	DOCK1	1793	dedicator of cytokinesis 1
-2.64	FH	2271	fumarate hydratase
-1.22	FHIT	2272	fragile histidine triad
1.07	FLCN	201163	folliculin
-5.48	FZR1	51343	fizzy/cell division cycle 20 related 1
-1.32	GTF2H1	2965	general transcription factor IIH, polypeptide 1
0.91	HDAC1	3065	histone deacetylase 1
-0.98	HDAC2	3066	histone deacetylase 2
0.17	HDAC6	10013	histone deacetylase 6
0.67	ING1	3621	inhibitor of growth family, member 1
-2.07	ING3	54556	inhibitor of growth family, member 3
-1.67	ING4	51147	inhibitor of growth family, member 4
-0.68	ING5	84289	inhibitor of growth family, member 5
-0.63	KAT2B	8850	K(lysine) acetyltransferase 2B
-0.18	LATS1	9113	large tumor suppressor kinase 1
-4.75	LLGL1	3996	lethal giant larvae homolog 1
-0.33	MAP3K4	4216	mitogen-activated protein kinase kinase kinase 4
1.44	MAPK1	5594	mitogen-activated protein kinase 1
-2.16	MAPK14	1432	mitogen-activated protein kinase 14
-0.10	MAPK3	5595	mitogen-activated protein kinase 3
-1.38	MCL1	4170	myeloid cell leukemia 1
0.65	MLH1	4292	mutL homolog 1
-1.00	MLL3	58508	myeloid/lymphoid or mixed-lineage leukemia 3
-0.63	MNAT1	4331	MNAT CDK-activating kinase assembly factor 1
-2.46	MSH2	4436	mutS homolog 2
-0.66	MSH6	2956	mutS homolog 6
0.11	MTIF2	4528	mitochondrial translational initiation factor 2
-1.54	MTOR	2475	mechanistic target of rapamycin
-0.63	NF1	4763	neurofibromin 1

0.54	NF2	4771	neurofibromin 2 (merlin)
-0.11	PALB2	79728	partner and localizer of BRCA2
0.62	PBRM1	55193	polybromo 1
-0.69	PHB2	11331	prohibitin 2
1.04	PMS2	5395	PMS2 postmeiotic segregation increased 2 (S. cerevisiae map)
-0.10	PPP2CB	5516	protein phosphatase 2, catalytic subunit, beta isozym
-0.25	PRKCA	5578	protein kinase C, alpha
-0.34	PRKCB1	5579	protein kinase C, beta
-1.48	PRKCG	5582	protein kinase C, gamma
-0.23	PSME4	23198	proteasome (prosome, macropain) activator subunit 4
1.57	PTEN	5728	phosphatase and tensin homolog
-5.53	RAD17	5884	RAD17 homolog (S. pombe)
-2.74	RAD23B	5887	RAD23 homolog B (S. cerevisiae map)
-0.17	RAD50	10111	RAD50 homolog (S. cerevisiae map)
1.27	RAD51	5888	RAD51 recombinase
0.45	RAD52	5893	RAD52 homolog (S. cerevisiae map)
-1.04	RB1	5925	retinoblastoma 1
-6.44	RFC1	5981	replication factor C (activator 1) 1
-0.59	RPL11	6135	ribosomal protein L11
-3.84	RPS10	6204	ribosomal protein S10
-1.98	RPS11	6205	ribosomal protein S11
-2.37	RPS19	6223	ribosomal protein S19
-0.08	RRM1	6240	ribonucleotide reductase M1
-0.23	SDHB	6390	succinate dehydrogenase complex, subunit B, iron sulfur
0.38	SETD2	29072	SET domain containing 2
-0.15	SH3GL1	6455	SH3-domain GRB2-like 1
0.22	SHPRH	257218	SNF2 histone linker PHD RING helicase
-0.66	SIN3A	25942	SIN3 transcription regulator family member A
-0.15	SMARCB1	6598	SWI/SNF related, matrix associated, actin dependent regulator of chromatin, subfamily b, member 1
0.02	SMARCC1	6599	SWI/SNF related, matrix associated, actin dependent regulator of chromatin, subfamily c, member 1
-1.60	STK11	6794	serine/threonine kinase 11
-0.35	TCEA1	6917	transcription elongation factor A (SII), 1
0.44	TFE3	7030	transcription factor binding to IGHM enhancer 3
1.30	TFEB	7942	transcription factor EB
1.76	TP53	7157	tumor protein p53
0.31	TPM1	7168	tropomyosin 1 (alpha)
1.00	TSC1	7248	tuberous sclerosis 1

-3.41	TTN	7273	titan
1.37	TUSC3	7991	tumor suppressor candidate 3
0.25	UBE4B	10277	Ubiquitin conjugation factor E4 B
-1.68	USP11	8237	ubiquitin specific peptidase 11
-0.32	VHL	7428	von Hippel-Lindau tumor suppressor
1.57	WASL	8976	Wiskott-Aldrich syndrome-like
-13.07	WEE1	7465	WEE1 G2 checkpoint kinase
-3.52	WRN	7486	Werner syndrome, RecQ helicase-like
-0.32	XPA	7507	xeroderma pigmentosum, complementation group A
-2.05	XRCC3	7517	X-ray repair complementing defective repair in Chinese hamster cells 3
0.77	XRCC4	7518	X-ray repair complementing defective repair in Chinese hamster cells 4
0.36	XRN1	54464	5'-3' exoribonuclease 1
-0.21	YWHAE	7531	tyrosine 3-monooxygenase/tryptophan 5-monooxygenase activation protein, epsilon
-0.80	YWHAZ	7534	tyrosine 3-monooxygenase/tryptophan 5-monooxygenase activation protein, zeta

Supplemental Table 2: *H. sapiens – S. cerevisiae* gene alignment scores using ClustalW

	cerevisiae RAD24	cerevisiae RAD17	human RAD17
cerevisiae RAD24	100		
cerevisiae RAD17	50.96	100	
human RAD17	55.15	65.24	100

	cerevisiae RAD53	cerevisiae DUN1	human CHEK2
cerevisiae RAD53	100		
cerevisiae DUN1	65.82	100	
human CHEK2	51.82	89.43	100

	cerevisiae CHK1	human CHEK1
cerevisiae CHK1	100	
human CHEK1	52.65	100

Supplemental Table 3: Relative shRAD17 abundance and *CHEK1/2* status for 102 cell lines tested in Project Achilles

Cell Line	relative shRAD17 abundance	CHEK1	CHEK2
GP2D_LARGE_INTESTINE	**0.394**	mut	
COLO704_OVARY	**0.504**		HOMDEL
LS411N_LARGE_INTESTINE	**0.544**	mut	
NCIH2171_LUNG	**0.601**		
LN319_CENTRAL_NERVOUS_SYSTEM	**0.634**		
PANC1005_PANCREAS	**0.667**	mut	
KM12_LARGE_INTESTINE	**0.706**	mut	mut
NCIH661_LUNG	**0.712**		
QGP1_PANCREAS	**0.714**		
OVCAR8_OVARY	**0.723**		
HL60_HAEMATOPOIETIC_AND_LYMPHOID_TISSUE	**0.732**		
CFPAC1_PANCREAS	**0.737**		
MIAPACA2_PANCREAS	**0.750**		
TE9_OESOPHAGUS	**0.751**		mut
A2780_OVARY	**0.763**		
COLO205_LARGE_INTESTINE	**0.767**		
HT29_LARGE_INTESTINE	**0.768**		
U251MG_CENTRAL_NERVOUS_SYSTEM	**0.779**		
KP4_PANCREAS	**0.786**		
CH157MN_CENTRAL_NERVOUS_SYSTEM	**0.789**		
SW48_LARGE_INTESTINE	**0.803**		
IGROV1_OVARY	**0.814**	mut	
KYSE450_OESOPHAGUS	**0.830**	HOMDEL	
COLO741_SKIN	**0.833**		
HCC827_LUNG	**0.835**		
PANC0813_PANCREAS	**0.836**		
KP1NL_PANCREAS	**0.838**		
SNUC2A_LARGE_INTESTINE	**0.839**		
SKCO1_LARGE_INTESTINE	**0.845**		
IOMMLEE_CENTRAL_NERVOUS_SYSTEM	**0.849**		
SLR21_KIDNEY	**0.851**		
LN464_CENTRAL_NERVOUS_SYSTEM	**0.851**		
COV504_OVARY	**0.853**		
SJSA1_BONE	**0.858**		
KMS12BM_HAEMATOPOIETIC_AND_LYMPHOID_TISSUE	**0.862**		
A204_SOFT_TISSUE	**0.869**		
TE15_OESOPHAGUS	**0.873**		
NCIH196_LUNG	**0.885**		HOMDEL
LN215_CENTRAL_NERVOUS_SYSTEM	**0.891**		

NIHOVCAR3_OVARY	**0.894**			
HCC364_LUNG	**0.894**			
HT55_LARGE_INTESTINE	**0.895**			
HCC2814_LUNG	**0.902**			
OE33_OESOPHAGUS	**0.903**			
JHESOAD1_OESOPHAGUS	**0.907**			
F5_CENTRAL_NERVOUS_SYSTEM	**0.911**			
LN229_CENTRAL_NERVOUS_SYSTEM	**0.922**			
EFO21_OVARY	**0.925**			
KYSE150_OESOPHAGUS	**0.927**			
TOV21G_OVARY	**0.934**			
HCC70_BREAST	**0.938**			
OVMANA_OVARY	**0.940**			
NCIH2122_LUNG	**0.955**			
HEYA8_OVARY	**0.961**			
TYKNU_OVARY	**0.972**			
KYSE30_OESOPHAGUS	**0.974**			
SNUC1_LARGE_INTESTINE	**0.978**			
RKO_LARGE_INTESTINE	**0.979**			
HEC1A_ENDOMETRIUM	**0.983**			
OVISE_OVARY	**0.984**			
COV434_OVARY	**0.990**			
RT112_URINARY_TRACT	**0.990**			
TOV112D_OVARY	**0.998**			
HPAC_PANCREAS	**1.007**			
A2058_SKIN	**1.010**			
PANC0327_PANCREAS	**1.011**			
786O_KIDNEY	**1.015**			
OV90_OVARY	**1.022**			
KYSE510_OESOPHAGUS	**1.026**			
HLF_LIVER	**1.040**			
SU8686_PANCREAS	**1.050**			
NCIH82_LUNG	**1.054**			
LOVO_LARGE_INTESTINE	**1.056**			
NCIH1975_LUNG	**1.058**			
A549_LUNG	**1.065**			
EFO27_OVARY	**1.071**			
DLD1_LARGE_INTESTINE	**1.077**			
JHOC5_OVARY	**1.086**			
HS944T_SKIN	**1.090**			
TT_OESOPHAGUS	**1.103**			
HUTU80_SMALL_INTESTINE	**1.117**			
KURAMOCHI_OVARY	**1.145**			
CAOV4_OVARY	**1.196**			

MDAMB453_BREAST	**1.196**		
RMGI_OVARY	**1.206**	HOMDEL	
LS513_LARGE_INTESTINE	**1.213**		
NCIH508_LARGE_INTESTINE	**1.215**		
C2BBE1_LARGE_INTESTINE	**1.221**		
SW480_LARGE_INTESTINE	**1.236**		
RKN_OVARY	**1.251**		
NCIH1650_LUNG	**1.270**		
BXPC3_PANCREAS	**1.293**		
L33_PANCREAS	**1.322**		
CAOV3_OVARY	**1.324**		
SF767_CENTRAL_NERVOUS_SYSTEM	**1.339**		
IGR39_SKIN	**1.380**		
SNU840_OVARY	**1.399**		
COV362_OVARY	**1.435**		
AGS_STOMACH	**1.449**		
OVCAR4_OVARY	**1.530**		
ASPC1_PANCREAS	**1.531**		
HUG1N_STOMACH	**1.741**		

nut = mutation

IOMDEL = HOMDEL

Supplemental Table 4: Summary of 2way ANOVA results for AZD7762, MK-1775, and combination.

Source of Variation by 2way ANOVA	% of total variation						
	AZD7762		MK-1775		AZD7762 + MK-1775		AZD7762 + VX-970
	HeLa	LN428	HeLa	LN428	HeLa	LN428	HeLa
Interaction	13.7	4.3	14.6	9.3	8.6	10.0	19.0
Dose of Drug	65.8	55.9	50.6	54.4	78.5	39.4	57.0
RAD17-KD	19.5	38.6	30.0	33.9	12.1	48.8	21.5

Source of Variation by 2way ANOVA	p value						
	AZD7762		MK-1775		AZD7762 + MK-1775		AZD7762 + VX-970
	HeLa	LN428	HeLa	LN428	HeLa	LN428	HeLa
Interaction	< 0.0001	< 0.0001	< 0.0001	< 0.0001	< 0.0001	< 0.0001	< 0.0001
Dose of Drug	< 0.0001	< 0.0001	< 0.0001	< 0.0001	< 0.0001	< 0.0001	< 0.0001
RAD17-KD	< 0.0001	< 0.0001	< 0.0001	< 0.0001	< 0.0001	< 0.0001	< 0.0001
